# Study on the Deterioration of Chloride Erosion in Tunnel Construction Joints Under a Multifactorial Environment

**DOI:** 10.3390/ma18122854

**Published:** 2025-06-17

**Authors:** Weiwei Han, Wenming Zhang, Shirong Guo, Ruifeng Nie, Weijie Zhang, Shuyin Wu

**Affiliations:** 1 College of Civil Engineering and Architecture, Shandong University of Science and Technology, Qingdao 266590, China; skd994504@sdust.edu.cn (W.H.); skd994549@sdust.edu.cn (R.N.);; 2 Qingdao Municipal Construction Group Co., Ltd., Qingdao 266112, China; tg98792025@163.com; 3 China Railway 22nd Bureau Group Co., Ltd., Beijing 100043, China; shirongguo_0430@126.com

**Keywords:** chloride, water pressure, erosion

## Abstract

Structural crack seepage in concrete is a common condition in engineering applications. Under the combined effects of multiple factors such as water pressure and load, cracks are more likely to occur inside the concrete structure, thus aggravating the water seepage problem. To simulate the chloride ion erosion of structural cracks, an independent test system that can simultaneously consider the coupling effect of multiple factors was developed. Three typical factors—water pressure, vertical load, and erosion time—were selected and designed using the orthogonal test method to analyze the effect of factors on the chloride ion concentration. The results revealed that the vertical load is the least influential factor, water pressure and erosion time are the most noticeable factors, and the factors influencing the diffusion of chloride ion in concrete are, in order of magnitude, water pressure (0.86), erosion time (0.66), and vertical load (0.36). Nonlinear surface fitting, with an R-squared value exceeding 0.95, was used to characterize the relationship between chloride ion concentration, water pressure, and erosion time.

## 1. Introduction

Concrete structures in service in marine environments are subjected to salt erosion and pressure, making the problem of substantial durability failure more prominent. Under the combined effects of water pressure and load, cracks are more likely to occur inside the concrete structure, thus aggravating the water seepage problem. Groundwater in coastal areas has high chloride salt concentrations. Chloride ions enter the concrete through concrete cracks. The hydration products of the cement chemical reaction generate calcium trichloroaluminate and other expansive substances, causing the concrete to lose and destroy the passivation film on the surface of the steel reinforcement, leading to rapid corrosion of the steel reinforcement and resulting in the rapid deterioration of the concrete structure.

In recent years, scholars have conducted numerous theoretical and engineering studies on chloride transport in concrete structures in marine environments. Liu established a simplified formula for the quantitative index of the chloride ion permeability of concrete based on the cumulative chloride ion content, considering the temperature and dry–wet cycles, two typical marine environmental factors [1]. Xiu et al. summarized the experimental methods for exploring chloride ion erosion in concrete in a marine salt spray environment, discussed the factors affecting chloride ion transport, and generalized a chloride ion transport model [2]. Zhao and Gai summarized the chloride ion erosion process of static and repeated loads, proposed a chloride ion transport mechanism in concrete under load, and illustrated the practical value of the erosion model for the structural durability of concrete bridges subjected to chloride salt erosion [3]. Ma et al. studied the transport characteristics of chloride ions at different hydrostatic pressures, mixing ratios, and pressure-action times, and found that under hydrostatic pressure, the chloride penetration depth and content increased with increasing hydrostatic pressure and action time [4]. Liu et al. deduced a chloride diffusion erosion model of concrete considering linearly distributed stresses and proved that the established chloride erosion model could reflect the effect of linearly distributed stresses on chloride ion diffusion in the structural cross section [5]. Jin and Wang investigated the diffusion of chloride ions in the protective layer of concrete and found that, at the same depth of the protective layer, as the load increases, the chloride ion content increases, and the corrosion rate of the reinforcement inside the concrete also increases [6]. Ribeiro et al. conducted chloride erosion tests by varying the concrete water–cement ratio, cement type, mortar content, and the amount of mineral additives. The chloride diffusion coefficients were corrected based on test data to improve the accuracy of the existing model [7].

Yu and Lin established a mesoscale finite element model for chloride ion transport within recycled concrete and analyzed the effects of factors such as the shape, location, substitution rate of recycled aggregate, and adhesion rate of old mortar on the transport of chloride ions within recycled concrete [8]. Zhu et al. investigated the diffusion of chloride ions in concrete structures under cyclic impact loading using a series of tests and concluded that cyclic impact load has a pronounced accelerating effect on chloride diffusion [9]. Pathan et al. presented a theoretical framework for modeling the service life of reinforced concrete structures in chloride environments using load factors and showed that the nature of the load (compression or tension) can lead to different diffusion results [10]. Kim et al. conducted field tests on in-service offshore structures and concluded that the dry/wet ratio significantly affects chloride transport in concrete, suggesting that, owing to the complexity of the marine environment, the results of exposure tests may be affected by various factors and thus exhibit randomness [11]. Wang et al. combined the Boltzmann–Matano method with measured chloride ion concentration data to establish a predictive model for the chloride ion diffusion coefficient in concrete, which can quantitatively analyze the variation rule of the chloride ion diffusion coefficient in concrete with exposure time and diffusion depth [12]. Leung and Hou assumed that the diffusion coefficients of chloride ions in cracks and crack widths are independent of each other and further proposed an empirical formula for the macroscopic equivalent diffusion coefficients of chloride ions in cracked concrete by simulating the diffusion process of chloride ions in concrete containing a single crack [13].

Bentz et al. investigated the effect of transverse cracking on the diffusion of chloride ions in concrete, adapted a predictive model using a graphical approach, and combined it with experimental data on saturated concrete to determine whether cement paste mixed with chloride ions plays a vital role in slowing the entry of chloride ions into the concrete [14]. Studies show that even small cracks (0.1–0.3 mm) can drastically increase diffusion coefficients, leading to premature corrosion initiation [15,16,17]. Homan et al. investigated the effect of moisture transport on chloride penetration in partially saturated concrete and modified the governing equation that describes the chloride transport in saturated concrete to account for the chloride movement induced by the moisture transport [18]. Alsheet et al. proposed a chloride binding model consisting of physical adsorption and chemical ion exchange and determined that chloride transport by diffusion was limited to the region near the exposed surface, while in the concrete bulk it was dominated by electromigration [19]. From a large amount of experimental data on the diffusion of chloride ions in concrete, Mangat and Molloy confirmed that the magnitude of the chloride diffusion coefficient is highly dependent on the exposure time of the concrete [20]. Tegguer et al. prepared microcracks by axial precompression of standard and high-performance concrete, detected the degree of concrete damage using ultrasonic pulse velocity, and detected the change in the chloride diffusion coefficient of standard and high-performance concrete after chloride erosion [21]. Rahman et al. investigated the effects of compressive stress-induced damage on chloride ion transport in concrete. This study demonstrated that the damage associated with ballast-induced stress significantly increased the diffusion of chloride ions in concrete [22]. Al-Kutti et al. proposed a multiphysics field formulation to increase and quantify the chloride diffusivity of concrete owing to damage, expressing the quantitative damage index in terms of the degradation of the modulus of elasticity of concrete [23].

The above studies analyzed a single external factor rather than performing a comprehensive analysis of multiple factors, such as the erosion time, crack width, water pressure, and vertical load. The lack of a comparative analysis of the different influencing factors has led to certain limitations in the study of chloride erosion. Therefore, this study independently developed a set of test device systems according to the actual service state of an underwater concrete structure that were composed of a load loading device, dynamic water pressure device, and salt solution collection device. The systems could simultaneously consider the coupling effect of three factors: water pressure, vertical load, and erosion time. The aim of this study is to investigate the water pressure, vertical load, and erosion time on the chloride corrosion of concrete and establish the relationship between the chloride concentration and these factors.

## 2. Materials and Methods

### 2.1. Chloride Erosion Modeling Test System

Most model test systems for studying chloride erosion are large in volume and mass, complex to operate, and cannot simultaneously provide the values of water pressure and vertical load. The independently designed model test system consists of three parts: a load-loading device, a dynamic water pressure device, and a solution collection device.

#### 2.1.1. Pneumatic Loading Device

**Figure 1:**
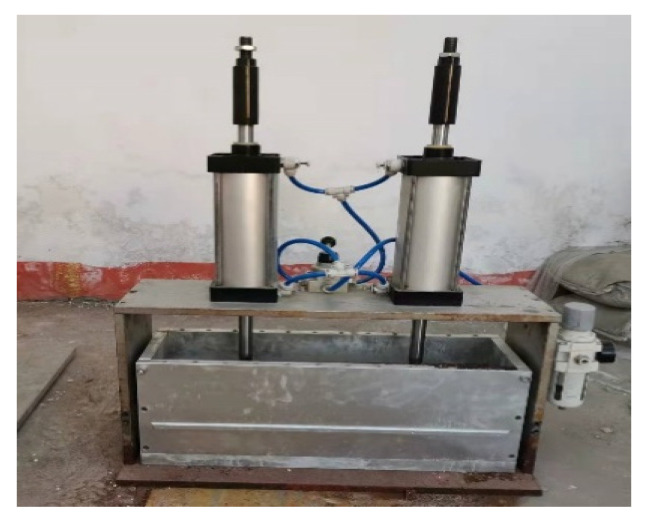
Loading cylinder.

The pneumatic loading devices (shown in Figure 1) include air compressors, loading cylinders, and reaction-frame compositions. The main working principle was to connect the air compressor to the pressure controller on the loading cylinder through a high-pressure pipe, and the pressure controller was connected to the cylinder through a high-pressure pipe. The pressure controller regulates the load applied by the cylinder to the concrete specimen. The reaction frame was connected to the base plate through bolts to provide the same counterforce as the cylinder loading and to stabilize the device.

#### 2.1.2. Water Pressure Loading Device

The hydraulic loading device comprised a variable-speed pump, pressurized water tank, water storage tank, and water pressure controller. First, the water pressure controller was set to the design pressure value, and the salt solution was pumped from the water storage tank to the pressurized water tank through the variable-speed pump. When the pressurized water tank reached the set pressure value of the variable-speed pump, it stopped working. When the pressure in the pressurized water tank decreased by 0.02 MPa, the variable-speed pump automatically started to extract the solution and convey it to the water tank.

#### 2.1.3. Erosion Test Block Model

The erosion specimen model comprised concrete specimens with cracks and steel molds connected by steel plates with bolts, with outer dimensions of 640 mm × 190 mm × 250 mm and with a water inlet and outlet at both ends. The size of the test block with cracks was 600 mm × 150 mm × 150 mm using layered casting.

#### 2.1.4. Crack Erosion Systems

**Figure 2:**
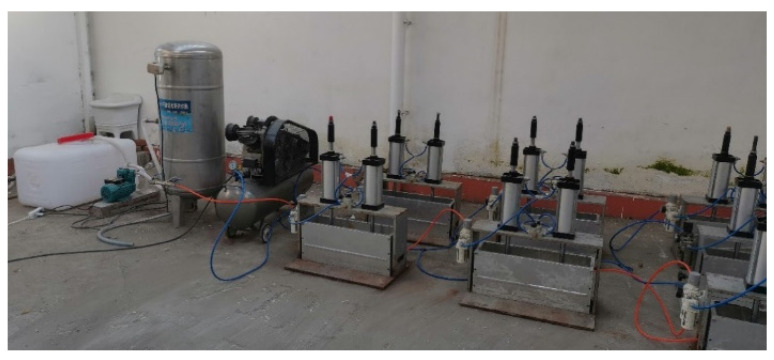
Fracture erosion modeling system.

**Figure 3:**
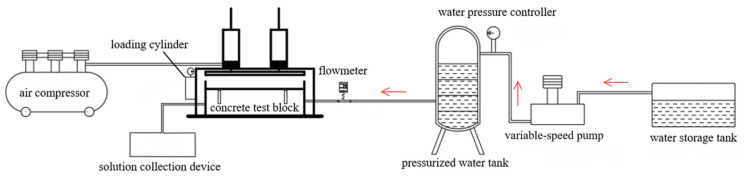
Schematic of the fracture seepage model system.

The complete crack erosion system consists of a combination of several parts of the test setup. Figure 2 and Figure 3 show the site picture and schematic, respectively.

### 2.2. Experimental Design

#### 2.2.1. Concrete Specimen Preparation

The specimen size was 600 mm × 150 mm × 150 mm, and layered pouring was used to prefabricate the artificial cracks, with the cracks in the specimen running in four steps.

The first step was to put the mixed concrete into a steel mold and place it on a vibrating table for vibration and compaction. The pouring height was 75 mm, which was exactly the middle height of the water inlet and outlet holes. After the beating was complete, it was placed in a curing room for 24 h, and the concrete was completely cured after mold removal and maintained for three days.

The second step was to use an electric hammer to slightly chisel the surface of the concrete, chisel off the surface cement film and loose stones, clean up, and sprinkle water for moistening.

The third step was brushing the cement mortar to ensure that the layered test blocks could be successfully separated. A small amount of fine sand was sprinkled on the prefabricated cracked cement mortar surface to conduct drilling powder testing.

The fourth step was to place the completed bottom specimen into the mold, pour 75 mm of postcast concrete, and vibrate it densely. The specimen was then placed in a curing room for 24 h. After 24 h of demolding, the two concrete test blocks were placed together in the curing room and cured under standard conditions for 28 d.

#### 2.2.2. Specimen Sealing

**Figure 4:**
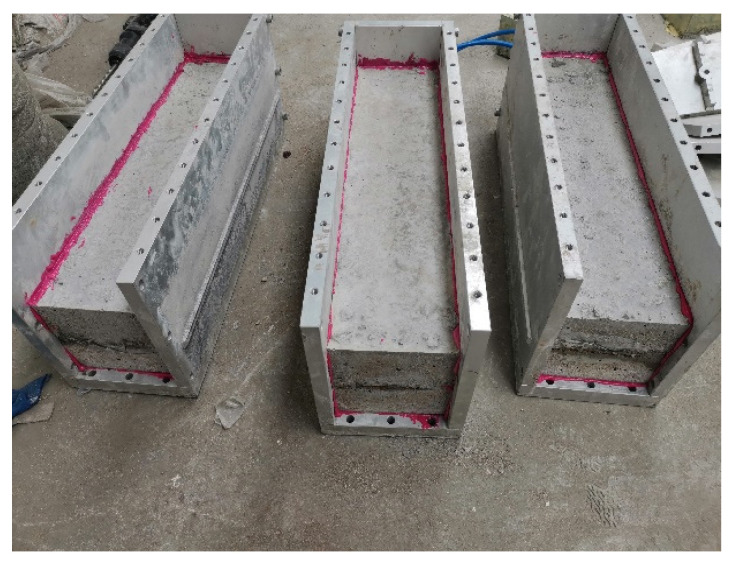
Specimen sealing.

To ensure that the chloride salt solution could smoothly pass through the prefabricated cracks and erode the crack surface, it was critical to ensure sealing between the specimen surface and inner wall of the mold. If the sealing does not meet the requirements, the salt solution will seep out along the side of the test block and the inner wall of the mold, the dynamic water pressure will not act on the crack surface, and the test will not achieve the desired results. Based on the literature and experimental methods, a process of sealing with the modified epoxy resin reinforcing adhesive was determined. First, the adhesive was applied to both sides of the crack for adhesive sealing to ensure passage of the salt solution through the gap. Subsequently, a closed rectangle was formed by applying the adhesive along the edge of the adhesive at the bottom. Finally, the steel plate was sealed completely and quickly to the side of the concrete test block, and the bolts were tightened as shown in Figure 4.

#### 2.2.3. Test Procedure

**Figure 5:**
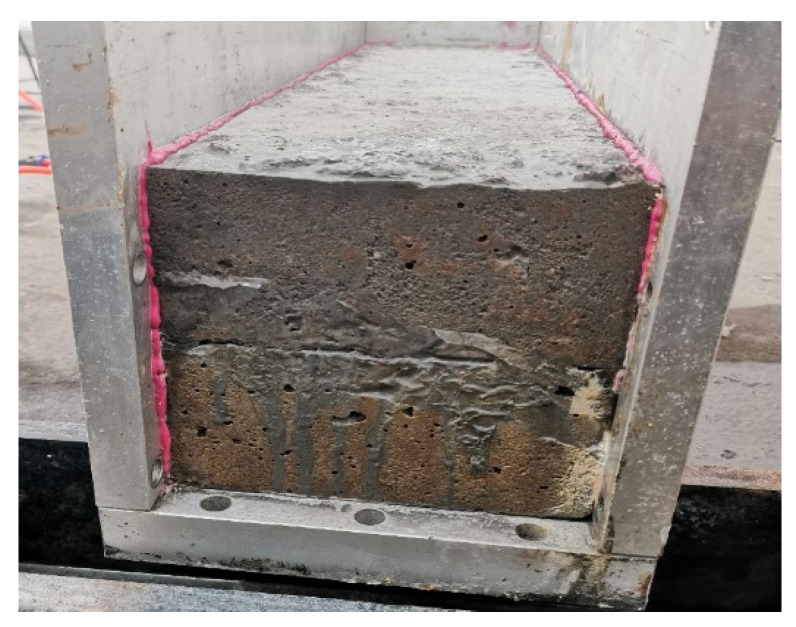
Salt solution seepage erosion.

The operation arrangement of the model test system is described below:

(1) Concrete was mixed according to the designed material and proportions, the mold was poured, and the concrete was cured under standard conditions.
(2) The concrete specimen was sealed with a mold after curing.
(3) The value of the water pressure controller on the pressurized water tank was set, and the frequency conversion water pump was turned on to extract the salt solution and convey it to the pressurized water tank; after reaching the set value, the water pump stopped automatically.
(4) The sealed test block and mold were placed on the base plate, and the position was fixed with a screw.
(5) The air compressor was connected to the cylinder, and the pressurized water tank was connected to the water inlet hole at the end of the mold.
(6) After the model system was connected, the lifting lever on the cylinder was adjusted for the vertical loading of the concrete specimen. Subsequently, the water flow and pressure were adjusted using a control valve.
(7) The time and flow rate of the water through the prefabricated cracks were observed to ensure that the salt solution seeped through the cracks into the water outlet. The salt solution was discharged into the designated collection box. Figure 5 shows crack seepage erosion.

#### 2.2.4. Experimental Program

For the multifactor test, the orthogonal experimental design was a simple and commonly used experimental design method. This test adopted an orthogonal practical approach combined with the specific conditions of the test for experimental design.

(1) Selection of test parameters

The salt solution concentration in this model system test was 7%, as this concentration is crucial for evaluating the durability of underwater structures against water pressure. Considering a water depth of 10 m and a corresponding water pressure of approximately 0.1 MPa, the test was designed to simulate three cases: water depths of 10 m, 20 m, and 30 m. For these three depths, the water pressure controller values were set to 0.1 MPa, 0.2 MPa, and 0.3 MPa, respectively. As the vertical load factors were applied to the lining cracks, the size of the applied axial pressure was set to 0 kN, 30 kN, and 60 kN at the three load levels. The design days for the erosion time of the chloride salt solution were 30 d, 60 d, and 90 d. Table 1 shows the detailed design of the program.

(2) Grouping

The test was based on an orthogonal test with three factors and three levels, and the trial was divided into nine groups. Table 2 shows the dimensions of the orthogonal test, delineated into three elements and three classes, and Table 3 shows the test grouping.

### 2.3. Sampling and Ion Content Determination

**Figure 6:**
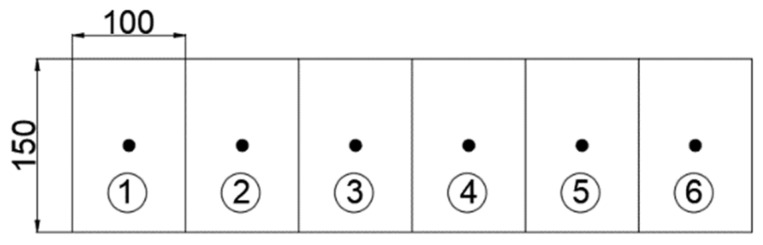
Schematic sampling location (mm).

The concrete sampling location in the length direction of the surface of the prefabricated cracks was divided into six cells, each of which was 100 mm × 150 mm. The concrete sample location was taken as the center of each cell, and the sampling depth was 10 mm. Figure 6 shows a schematic of the sampling location.

## 3. Results and Discussion

### 3.1. Determination of Ion Concentration

A rapid chlorine ion detector was used to determine the chloride concentrations. The results are shown in Table 4.

### 3.2. Range Analysis and Variance Analysis for Chloride Ion Content

Range analysis was applied to analyze the chloride ion concentration in the concrete based on orthogonal tests. Table 5 and Table 6 show the results of the analyses. Range value (R value) is the difference between the maximum k value and the minimum k value of for each factor. The larger the R value, the more significant the impact of the factor.

(1) Orthogonal analysis of the effect of water pressure on chloride ion concentration

**Figure 7:**
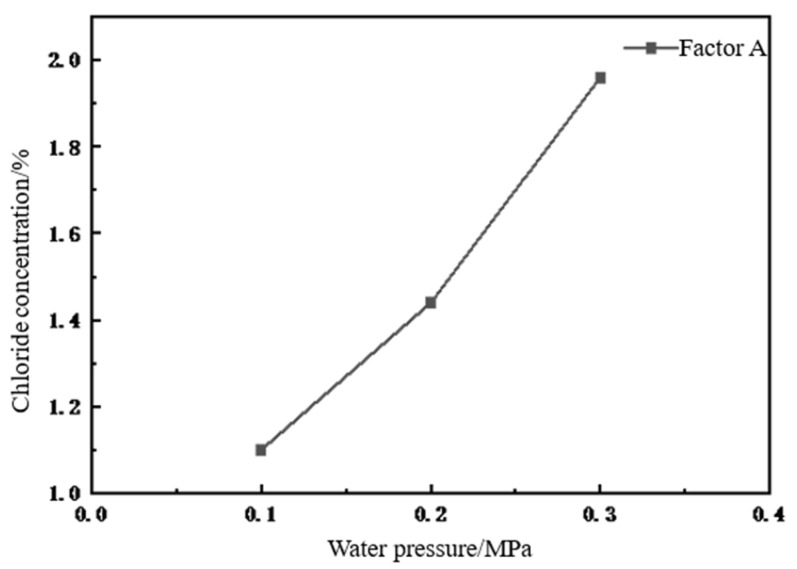
Relationship between water pressure and chloride ion concentration.

As shown in Figure 7, the maximum chloride ion concentration detected in the concrete specimens under a water pressure was Level 3 when a water pressure of 0.3 was applied. From Level 1 to Level 3, the chloride ion concentration increased by 30.9% and 36.1%, respectively, indicating that the water pressure had a significant effect on the diffusion of chloride ions in concrete. Thus, it can be assumed that water pressure accelerates the diffusion of chloride ions in concrete, which aggravates the erosion damage at concrete cracks.

(2) Effect of vertical loading on chloride ion concentration using orthogonal analysis

**Figure 8:**
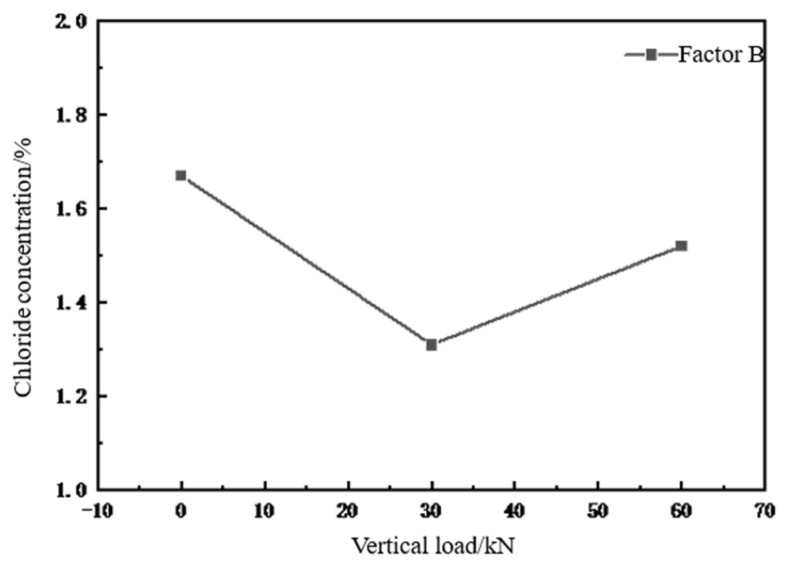
Relationship between uniaxial compressive load and chloride ion concentration.

As shown in Figure 8, the optimal value of the chloride ion concentration in the concrete specimens under a continuous vertical load was Level 1; that is, the load size was 0 kN; and the worst level value was level 2; that is, the load size was 30 kN. Without a compressive load, the chloride ion salt solution can rapidly diffuse into the concrete under a vertical pressure. After applying a minor stress to the concrete, the internal microcracks were inhibited or expanded. Chloride does not easily penetrate and diffuse, and concrete does not easily erode, making it difficult for chloride ions to enter the interior. When the vertical load increases to 60 kN, under the action of compressive stress, some of the micropores inside the concrete penetrated and promoted the diffusion of chloride ions, which accelerated the transmission rate of chloride ions in the concrete and accelerated the erosion damage process at the concrete cracks.

(3) Effect of erosion time on chloride ion concentration using orthogonal analysis

**Figure 9:**
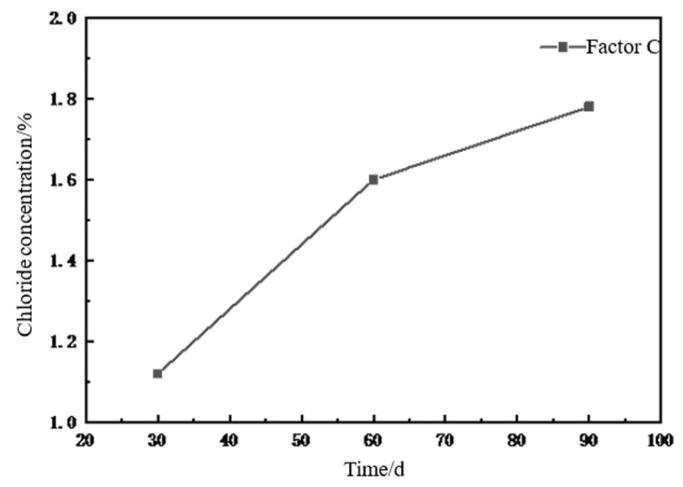
Relationship between erosion time and chloride ion concentration.

As shown in Figure 9, the chloride ion concentration in the concrete increased with the erosion time. The chloride ion concentration increased by 42.9% from 30 d to 60 d of erosion, whereas it increased by 11.2% from 60 d to 90 d. The increase in chloride ion concentration from 30 d to 60 d was more significant than that from 60 d to 90 d. The chloride ion content of concrete increased with increasing erosion time during the process of chloride salt erosion; however, the rate of growth of the ion content decreased, which indicated that the deterioration in concrete performance under chloride salt erosion increased with increasing erosion time. However, the rate of decline decreased with erosion time.

The results of the analysis above reveal that the water pressure, erosion time, and vertical load are all related to the diffusion of chloride ions in concrete. However, the degree of influence of each factor differs. The results of the calculated data within the test reveal the impact of three-factor level changes on the diffusion of chloride ions in concrete. Ranked in order of the magnitude of the extreme difference, the factors are as follows: water pressure (0.86) > erosion time (0.66) > vertical load (0.36). The most significant degree of influence on the erosion of chlorine salts was the water pressure, which promoted the diffusion of chloride ions the greatest, leading to a significant increase in the concentration of chloride ions, erosion time, and finally the axial pressure load factor.

Variance analysis was applied to analyze the chloride ion concentration, with blank columns as error groups. Table 7 and Table 8 show the detailed results. The critical value of the F-test is the minimum value that the F value must reach at a given level of significance to indicate a significant difference in variance between the sample groups. The significance level is usually set at three levels: 0.01, 0.05, and 0.1. If the F value exceeds the critical value (such as the 0.05 significance level), the result is significant.

Factor A was significantly associated with Factor C, Factor B was not significant, and the order of factor primacy was A–C–B. The most unfavorable combination was A3B1C3; that is, the water pressure and erosion time were the greatest. When there was no externally applied load, the concentration of chloride ions in the concrete was the greatest, and the chloride salts most severely eroded it.

### 3.3. Surface Fitting

**Figure 10:**
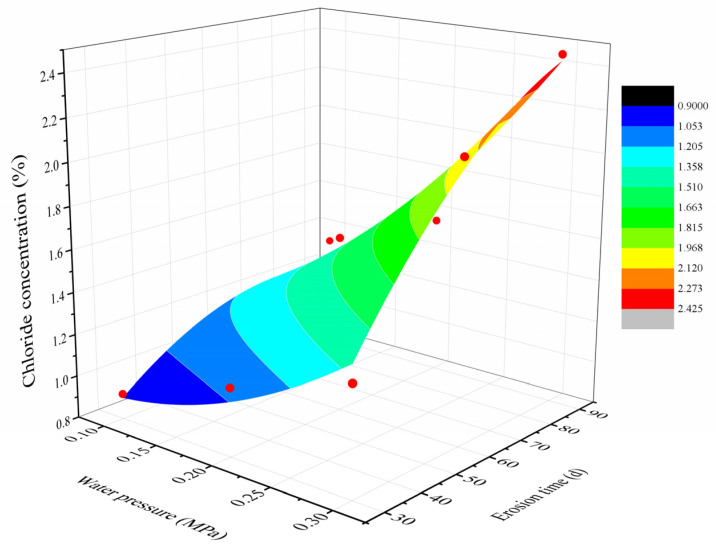
Relationship between chloride ion concentration and water pressure and erosion time (Origin surface fitting).

To verify the conclusion that factors A (water pressure) and C (erosion time) were significant and factor B (vertical load) was not, factor B was removed during data processing. Nonlinear surface fitting was performed on the test results corresponding to factors A and C using Origin software version 9.0, as shown in Figure 10, where the water pressure and erosion time are independent variables, and the chloride ion concentration of each erosion time of concrete specimen is the dependent variable. After multiple attempts, Equation (1) is the resulting function form with the largest R-squared, indicating that the fitting is the best:

z = z_0_ + a·x + b·y + c·x^2^ + d·y^2^ + e·x·y (1)

Continuing to analyze the fit yields z_0_ = 0.519; a = −3.033; b = 0.019; c = 0.061; d = 9.167; e = 0.00016; and R^2^ = 0.9567. The final fit can be expressed as Equation (2):

C = 0.519 − 3.033P + 0.019T + 0.061P^2^ − 9.167T^2^ − 0.00016P·T (2)

where C is the chloride ion concentration (%); P is the water pressure (MPa); and T is the erosion time (d).

The two-factor design values from the test were substituted into the surface fitting formula and compared with the test results to analyze the differences. Table 9 shows the specific results. The return value in the table is calculated with Equation (2).

Figure 10 and Table 9 show that the effect of vertical loading on the chloride ion content in concrete is negligible. The fitting results are similar to the experimental results; therefore, it is possible to study the relationship between the two factors, namely, the water pressure and erosion time, and the chloride ion content in concrete.

## 4. Practical Implications and Limitations

The test system developed in this study can be applied in practical engineering to help researchers gain a deeper understanding of the performance changes of concrete under conditions with water pressure, vertical load, and erosion time. The mathematical model established through nonlinear fitting can be used for performance prediction in practical engineering.

The current testing equipment is difficult to accurately simulate more complex multiple-factor-coupled environments such as high temperatures and chemical corrosion. The monitoring of the microstructure and the internal damage evolution process is also needed. At the same time, in-depth research on the interaction mechanism between factors should be conducted to establish more accurate mathematical models to describe the changes in concrete performance.

## 5. Conclusions

The chloride ion erosion of structural cracks was simulated in this study using an independent test system that can simultaneously consider the coupling effect of multiple factors. The experimental program was designed as an orthogonal test that considered three variables simultaneously: water pressure, vertical load, and erosion time. The tests were divided into nine groups based on a three-factor three-level orthogonal table. After the erosion process by the chloride salt solution was completed, the chloride ion concentration was determined and analyzed for each group. The conclusions are as follows:

(1) The range analysis of chloride concentrations measured in each group revealed that the following factors influenced the diffusion of chloride ions in concrete in order of magnitude: water pressure (0.86), erosion time (0.66), and vertical loading (0.36).
(2) The variance analysis revealed that Factor A (water pressure) and Factor C (erosion time) were significant and Factor B (vertical load) was not significant. When the water pressure and erosion time were the largest, and the externally applied load was the smallest, the concentration of chloride ions in the concrete was the largest, and erosion by the chloride salts was the most severe.
(3) A comparison of the results of the surface fitting of Factor A (water pressure) to Factor C (erosion time) against the experimental values revealed a slight difference, verifying that Factor A and Factor C were significant and Factor B was insignificant.

## Figures and Tables

**Table 1:** Orthogonal experimental design values.

Factors	Water Pressure/MPa	Vertical Load/kN	Erosion Time/d
Design values	0.1	0	30
	0.2	30	60
	0.3	60	90

**Table 2:** Orthogonal test factors and level values.

Level	Factors		
	Water Pressure/MPa	Vertical Load/kN	Erosion Time/d
1	0.1	0	30
2	0.2	30	60
3	0.3	60	90

**Table 3:** Three-factor and three-level orthogonal table.

Testing No.	A	B	C	Water Pressure/MPa	Vertical Load/kN	Erosion Time/d	Results/y_i_
1	1	1	1	0.1	0	30	y_1_
2	1	2	2	0.1	30	60	y_2_
3	1	3	3	0.1	60	90	y_3_
4	2	1	2	0.2	0	60	y_4_
5	2	2	3	0.2	30	90	y_5_
6	2	3	1	0.2	60	30	y_6_
7	3	1	3	0.3	0	90	y_7_
8	3	2	1	0.3	30	30	y_8_
9	3	3	2	0.3	60	60	y_9_

**Table 4:** Ion concentration results of each group under the orthogonal test.

Testing No.	Average Vertical Ion Concentration/%	Test No.	Average Vertical Ion Concentration/%	Test No.	Average Vertical Ion Concentration/%
1	0.92	4	1.63	7	2.45
2	1.05	5	1.56	8	1.31
3	1.33	6	1.12	9	2.11

**Table 5:** Calculation of the chloride ion concentration range.

Testing No.	Factors		
	A (Water Pressure)	B (Vertical Load)	C (Erosion Time)
1	1 (0.1 MPa)	1 (0 kN)	1 (30 d)
2	1	2 (30 kN)	2 (60 d)
3	1	3 (60 kN)	3 (90 d)
4	2 (0.2 MPa)	1	2
5	2	2	3
6	2	3	1
7	3 (0.3 MPa)	1	3
8	3	2	1
9	3	3	2
K1	3.3	5	3.35
K2	4.31	3.92	4.79
K3	5.87	4.56	5.34
k1	1.1	1.67	1.12
k2	1.44	1.31	1.6
k3	1.96	1.52	1.78
R	0.86	0.36	0.66

**Table 6:** Range analysis of the chloride ion concentrations for different factors.

Factors	Level Value of Each Factor			Range	Optimum Level	Bottom Level
	Level 1	Level 2	Level 3			
A (Water pressure/MPa)	1.1	1.44	1.96	0.86	3	1
B (Vertical load/kN)	1.67	1.31	1.52	0.36	1	2
C (Erosion time/d)	1.12	1.6	1.78	0.66	3	1

**Table 7:** Calculation of chloride ion concentration variance for different factors.

Testing Number	Factors			
	A (Water Pressure)	B (Vertical Load)	C (Erosion Time)	Blank Column
1	1 (0.1 MPa)	1 (0 kN)	1 (30 d)	1
2	1	2 (30 kN)	1	2
3	1	3 (60 kN)	1	3
4	2 (0.2 MPa)	1	2 (60 d)	3
5	2	2	2	1
6	2	3	2	2
7	3 (0.3 MPa)	1	3 (90 d)	2
8	3	2	3	3
9	3	3	3	1
K_1j_	3.3	5	3.35	4.59
K_2j_	4.31	3.92	4.79	4.62
K_3j_	5.87	4.56	5.34	4.27
K_1j_^2^	10.89	25	11.22	21.07
K_2j_^2^	18.58	15.37	22.94	21.34
K_3j_^2^	34.46	20.79	28.52	18.23

**Table 8:** Analysis of variance of chloride ion concentration for different factors.

Factors	Sum of Squared Deviations SS	Degrees of Freedom	Mean Square	F	F_0.01_(2,2)	F_0.05_(2,2)	F_0.1_(2,2)	Significance
A	1.12	2	0.56	56	99.0	19.0	9.0	Significant
B	0.2	2	0.1	10	99.0	19.0	9.0	
C	0.7	2	0.35	35	99.0	19.0	9.0	Significant
Error E	0.02	2	0.01					

**Table 9:** Origin surface fitting value compared with the measured value.

Testing No.	1	2	3	4	5	6	7	8	9
Test value	0.92	1.05	1.33	1.63	1.56	1.12	2.45	1.31	2.11
Return value	0.92	1.23	1.27	1.58	1.79	1.07	2.49	1.4	2.1
Difference in value	0	−0.18	0.06	0.05	−0.23	0.05	−0.04	−0.09	0.01
Variance rate/%	0	−17.1	4.5	3.1	14.7	4.5	−1.6	−6.8	0.4

## Data Availability

The original contributions presented in this study are included in the article. Further inquiries can be directed to the corresponding author.
